# Directing solar photons to sustainably meet food, energy, and water needs

**DOI:** 10.1038/s41598-017-03437-x

**Published:** 2017-06-09

**Authors:** Emre Gençer, Caleb Miskin, Xingshu Sun, M. Ryyan Khan, Peter Bermel, M. Ashraf Alam, Rakesh Agrawal

**Affiliations:** 1 Davidson School of Chemical Engineering, Purdue University, West Lafayette, IN 47907 USA; 2 School of Electrical and Computer Engineering, Purdue University, West Lafayette, IN 47907 USA

## Abstract

As we approach a “Full Earth” of over ten billion people within the next century, unprecedented demands will be placed on food, energy and water (FEW) supplies. The grand challenge before us is to sustainably meet humanity’s FEW needs using scarcer resources. To overcome this challenge, we propose the utilization of the entire solar spectrum by redirecting solar photons to maximize FEW production from a given land area. We present novel solar spectrum unbundling FEW systems (SUFEWS), which can meet FEW needs *locally* while reducing the overall environmental impact of meeting these needs. The ability to meet FEW needs locally is critical, as significant population growth is expected in less-developed areas of the world. The proposed system presents a solution to harness the same amount of solar products (crops, electricity, and purified water) that could otherwise require ~60% more land if SUFEWS were not used—a major step for Full Earth preparedness.

## Introduction

The world is expected to grow from seven to more than ten billion people, resulting in a “Full Earth” over the next century^1^. This increase in population coupled with rising per capita income and associated change in consumption habits will put unprecedented stress on food, energy and water (FEW) resources^1^. The grand challenge before us is to sustainably meet humanity’s FEW needs on a Full Earth using scarcer resources. The sun is the key energy source that can sustainably meet humanity’s FEW needs now and in the future. In light of this, we have developed novel solar spectrum unbundling FEW systems (SUFEWS), which meet *local* FEW needs for any foreseeable future, while reducing the overall environmental impact of meeting these needs.

**Figure 1:**
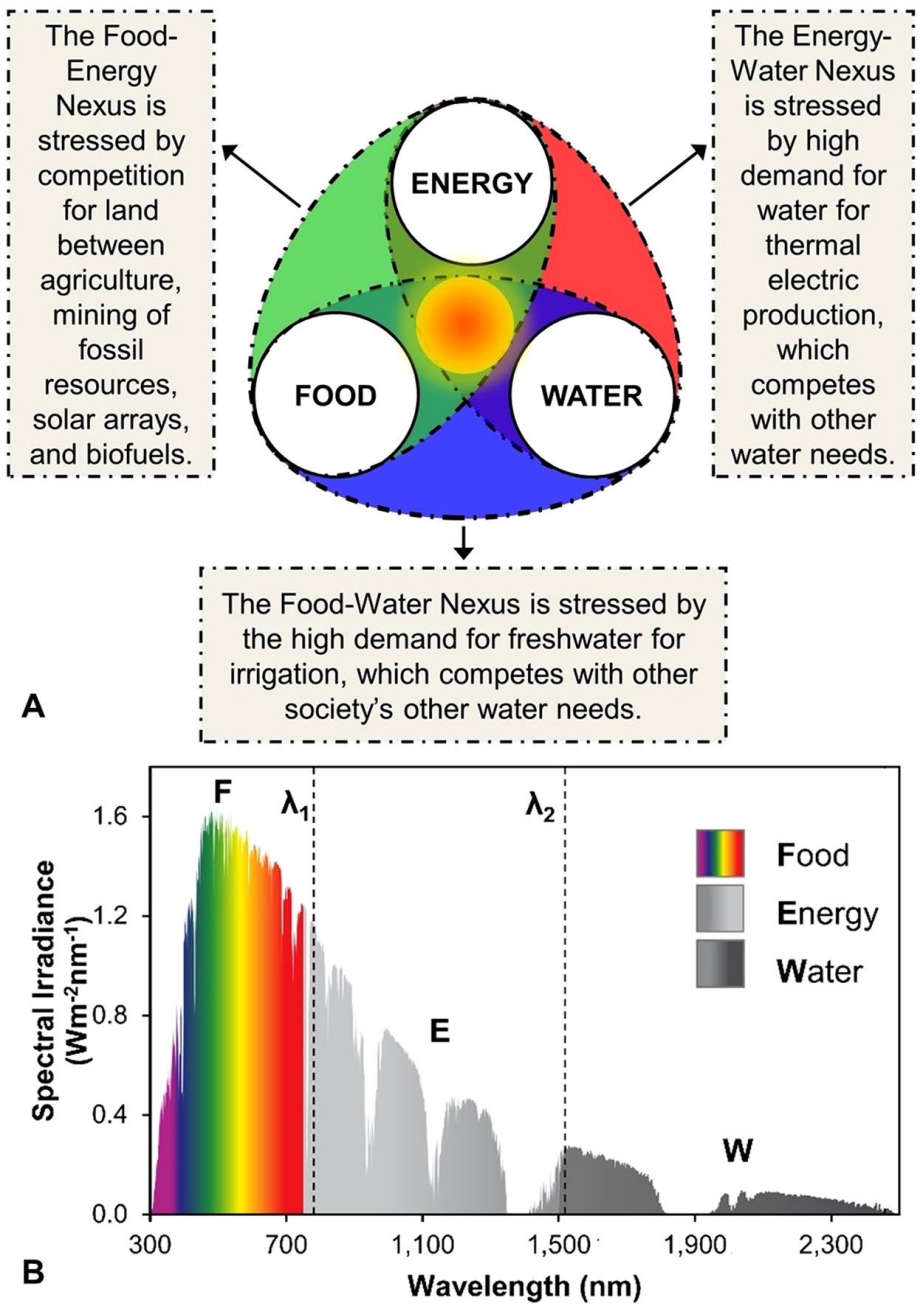
(**A**) Binary Food, Energy and Water nexus compounded in the FEW nexus. (**B**) AM1.5 G solar irradiance spectrum divided into three regions according to their nominal wavelength λ; F = λ < λ_1_, E = λ_1_ ≤ λ ≤ λ_2_, W = λ_2_ ≤ λ ≤ λ_3_. For our study λ_1_ = 750 nm.

Although the production of FEW resources can be highly connected, current practice mostly focuses on addressing individual or binary combinations of the FEW nexus. Each binary nexus within the FEW nexus has its own challenges that are compounded in the system as a whole. In the food-water nexus, global agriculture accounts for 75 to 86% of humanity’s consumptive water use^2, 3^. In competition with this is the energy-water nexus, which uses great quantities of water for hydropower, thermal electric plants, biofuel production, and oil and gas extraction via fracking^4^. In the food-energy nexus, the entire incident solar energy on a land area is dedicated towards growing food, with a majority of the energy of the incident photons being wasted or used inefficiently. The food-energy nexus is further stressed by the production of biofuels. These competing demands are considered jointly as the FEW nexus (Fig. 1A).

For a sustainable FEW nexus that supports the Full Earth, solar radiation is the sole energy source that is globally available and can meet energy needs^5–9^. However, the current practice is to use incident solar energy on a land area for only a single dedicated application. For example, due to strong shadow casted on the ground by current PV modules^10^, land areas dedicated to electricity generation that use typical PV or solar thermal installations cannot be simultaneously used for food production. When compared to adjoining shadow-free land areas, regions underneath the PV arrays receive 92% lower photosynthetically active radiation and grow only one-fourth of the biomass^11^. As a result, low energy photons below the band gap on a PV farm, as well as the majority of photons outside the photosynthetic range on a farmland, all go unused. Furthermore, solar energy is only passively used via the traditional water cycle (i.e. evaporation, condensation, rain) and not directly used to treat water locally at agricultural and urban centers for water management, purification, and recycling. Land availability will only be increasingly constrained as earth’s population increases, especially when urban and agriculture centers are co-located. Next generation PV technologies such as organic PVs^12^ and perovskite solar cells^13^ can enable tandem applications. While building and vehicle integrated PV systems are other promising methods to increase PV output from land areas devoted to other uses^14^, which may benefit from a variety of emerging, low-cost materials, modeling their output and impact is beyond the scope of this paper.

Although in theory, harnessing solar energy from 1–2% of the earth’s land area can meet global energy needs, when one compares local solar intensity with local energy demand in locations where the humans live, it is clear much more land will be necessary. Estimates show that averaged over the entire year, PV parks in northern Europe deliver 4–5 W/m^2^, whereas Britain and Germany’s rate of energy consumption based on total land area in each country is 1.25 W/m^2^ ^15^. Therefore, in a solar-powered world economy, the land area needed to meet the local energy need will often be an order of magnitude higher than the commonly used number of ~2%^15^.

Solar spectrum splitting to maximize electric power generation and heat recovery is well known^16^. However, the spectrum splitting and its feasibility in the context of all three FEW elements from the same land has never been reported. Here, we not only consider this possibility and develop systems to achieve this goal but also through modeling show the vast unexplored potential of such a system towards meeting FEW needs for a full earth. We present a novel system that enables the efficient use of the full solar spectrum and allows for FEW production from the same unit area of land. As shown in Fig. 1B, our system starts with the premise that the solar spectrum can be shared within the FEW nexus to eliminate competing demands among food production, electric power generation, and water purification/recycling. Since virtually all C3 and C4 crops use photons within the wavelength range of about 350 to 750 nm^17^, we unbundle the solar spectrum into three distinct regions: F for food production (λ ≤ 750 nm), E for electricity generation (750 nm < λ < λ_2_), and W for water purification (λ ≥ λ_2_) as shown in Fig. 1B. The value of λ_2_ is a system optimization parameter as discussed later.

## Proposed SUFEWS system

**Figure 2:**
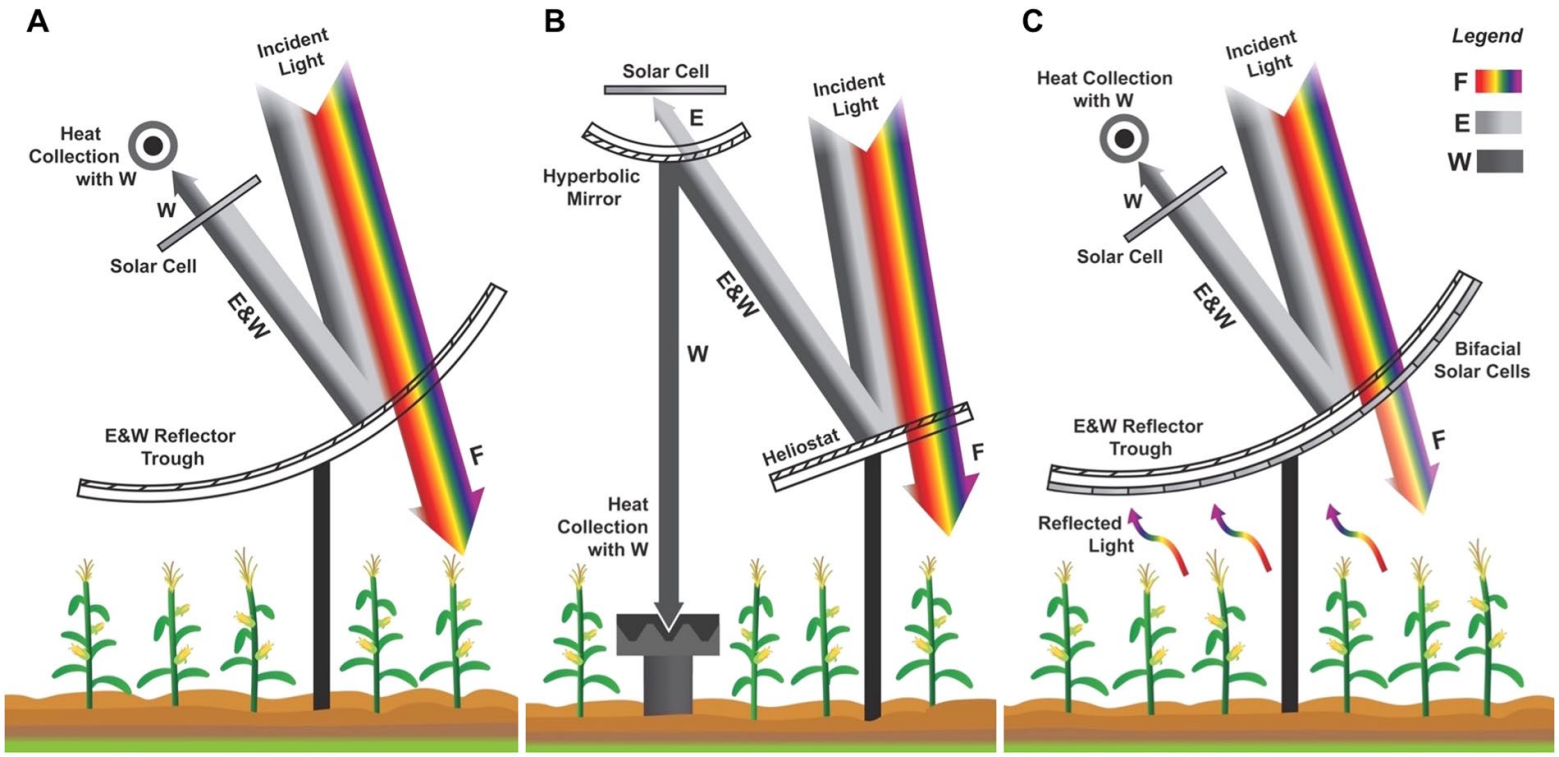
Illustration of the solar photons unbundling concept for solar photons through three alternative arrangements: (**A**) A parabolic trough with a reflective surface that transmits full intensity F (scenario A) for plant growth and reflects E&W. The solar cell absorbs E and transmits W for heat collection to purify water by multistage flash or multieffect distillation. (**B**) A heliostat with the same reflective surface as in A, but W is reflected off of a hyperbolic mirror to a heat cavity before E is incident on solar cells. (**C**) For plants that can thrive in a reduced intensity of F (scenario B), the same arrangement as A, but a thin bifacial solar cell is used under the reflective surface to harness a portion of F for electricity generation and any reflected light from underneath the trough. (Figure credit: Ryan Ellis, Purdue University).

Our basic concept for SUFEWS is illustrated in Fig. 2. Reflector troughs or heliostats are situated above cropland to allow for agricultural activities. These are coated using well-studied methods^16, 18, 19^ such that the F spectrum passes through while all other photons are directed for further use. In design 1 (Fig. 2A), the E and W portions of the spectrum are directed to a solar cell of band gap equivalent to λ_2_ with a transparent back contact that allows W to be collected for heat. In design 2 (Fig. 2B), the W portion is reflected using a hyperbolic mirror back to a collector at ground level, while the E spectrum passes through to a solar cell. Design 3 (Fig. 2C) is similar to design 1, except that bifacial solar cells are used on the back of the troughs. The thickness of these cells or the density of their coverage on the troughs can be adjusted depending on the crops’ tolerance to reduced sunlight intensity. When the bifacial cells do not cover the entire reflector, simple light diffusers can be used on the remaining area of the reflector to avoid shading. Bifacial solar cells offer the additional advantage of collecting light scattered back from the ground and crops^20^. Of course, many other variations on these designs can be imagined (e.g. bifacial solar cells could be added to design 2). As in existing solar farm practices, empty spaces will be left between the arrays of parabolic trough or heliostats, so only about half the land is covered with these units. These designs allow for the use of agricultural land to simultaneously grow and transport food, generate electricity, and provide heat/electricity for water purification.

**Figure 3:**
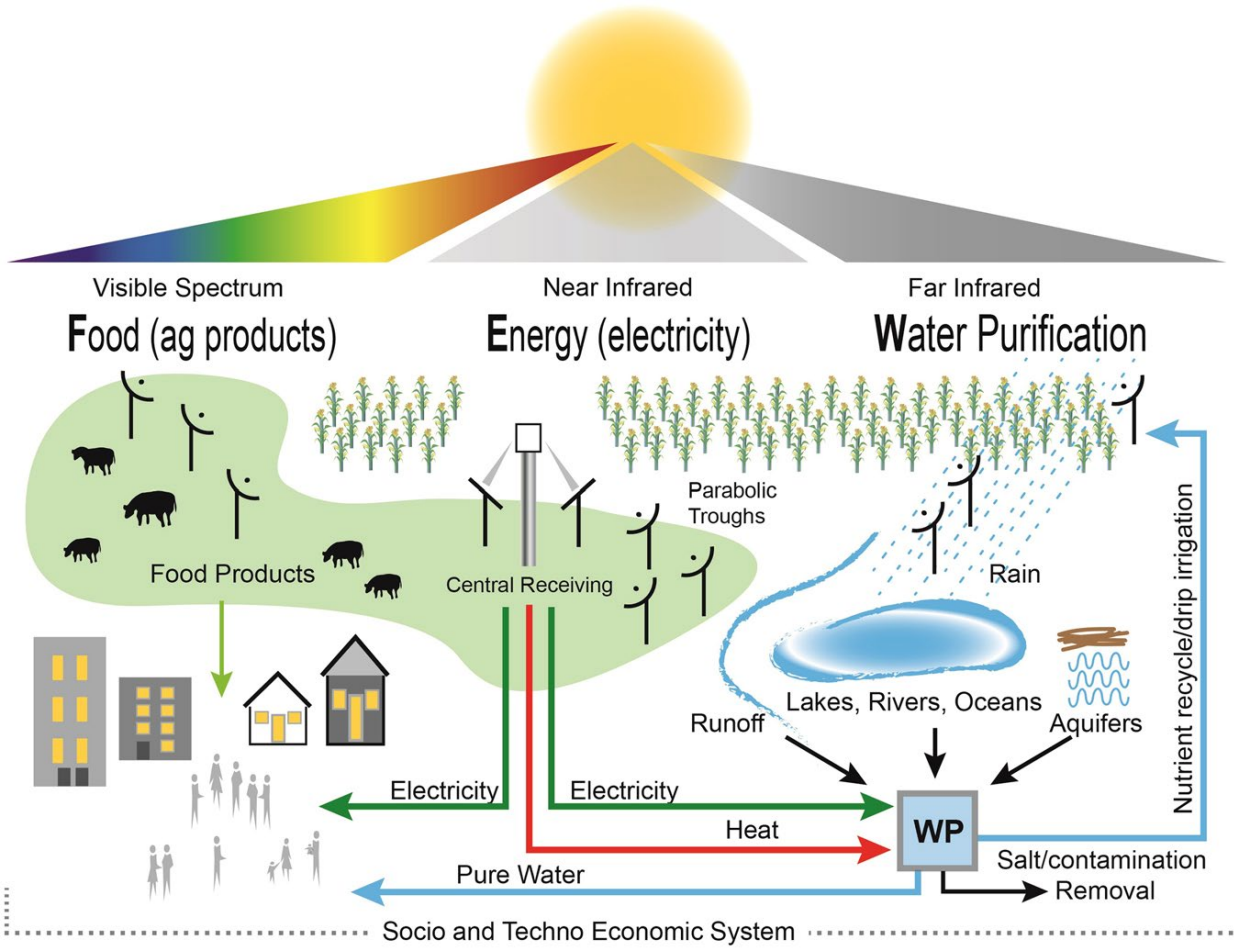
Conceptual implementation of SUFEWS in which photons are managed efficiently over crop/pasture land to simultaneously and harmoniously produce FEW products in a sustainable future for a Full Earth. (Figure credit: Pamela Burroff-Murr, Purdue University).

Our system-level concept is illustrated in Fig. 3. Water from sources such as underground, ocean, river, lake, ponds, and field runoff enter water purification (WP) units, which are powered by the heat and electricity generated as shown in Fig. 2. The purified water is then used for irrigation and urban needs. The salt or contamination-rich water leaving the WP units is sent for further processing/recycling/disposal. Similarly, electricity generated is used for agricultural production, with the excess being exported for use in population centers. Meanwhile, the supply of food products is unaffected.

Two distinct technologies are available for water purification and desalination: one uses heat for a multistage flash (MSF) shown in Fig. S4 or a multi effect distillation (MED), and the other uses electricity via membrane-based technologies such as reverse-osmosis (RO)^21–26^. Membrane-based technologies would eliminate the need to recover heat and will be simpler and potentially more cost-effective to implement for small projects, due to elimination of heat collection equipment; however, overall solar photon usage will be less efficient as W photons are not harvested. Detailed process simulation is only performed for MSF desalination using the Aspen Plus program (see SI section 4 for details). Various RO desalination approaches could also be considered for greater fresh water supply needs.

A major feature of SUFEWS is the ability to produce FEW resources locally without interfering with agricultural production, which will be increasingly important, as expected population growth will require increased dedication of land resources to agriculture. Note that the top 3 contributors to future world population growth, India, Nigeria, and Pakistan, already dedicate 60.6, 77.7, and 47.1 percent of their land to agriculture^27^. Additionally, much of the world’s expected population growth will occur in developing nations^28^ where the lack of infrastructure will greatly benefit from the local, distributed production of FEW goods that SUFEWS offers. The local generation of electricity will allow use of microgrids in villages and provide new paradigm for electricity generation and distribution. Local generation of power and clean water is also expected to reduce the long distance transmission losses inherent in any power supply grid system.

## Results and Discussion

In order to assess the feasibility of SUFEWS, we have designed, modeled, and optimized the entire system for FEW production for the arrangement in Fig. 2A using the AM 1.5D spectrum. First, we calculated the band gaps of single junction (SJ) and double junction (DJ) tandem solar cells that will give maximum efficiency of power conversion for the remaining photons after the F portion of spectrum has been subtracted at various solar concentrations assuming only radiative recombination in solar cells. The results for maximum power are shown in Fig. S3 with the F portion terminating at λ_1_ = 750 nm (1.65 eV, also see Fig. 1B). We calculated the optimal band gap for a SJ solar cell to be 0.928 eV (λ_2_ = 1340 nm). For a DJ cell, the optimal band gap for the top cell is ~1.14 eV and the bottom cell is 0.7 eV (λ_2_ = 1780 nm). For SJ cell case, all photons with energy less than ~0.928 eV belong to W photons and were harnessed as heat for water purification using our simulation model for MSF desalination (see Table S1 in Electronic Supplementary Information for energy content in split portions of F, E and W for each case).

**Figure 4:**
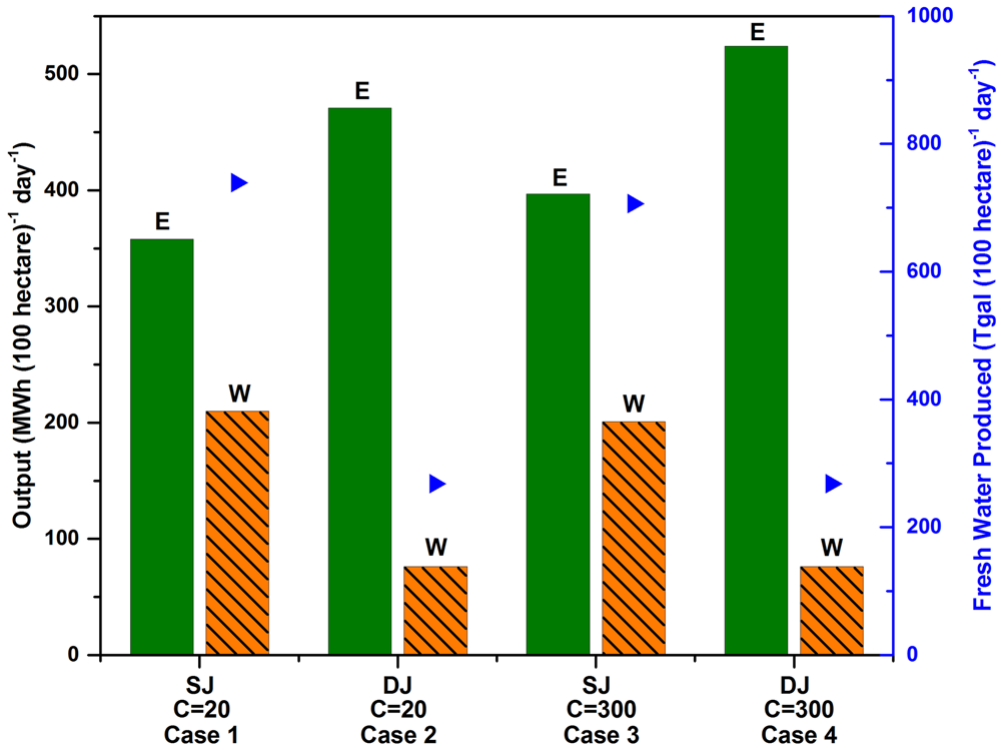
Electricity generated (**E**) and heat recovered (**W**) along with the fresh water produced (indicated by the arrows) from the recovered heat per 100 hectare of land for two solar concentration cases of 20 and 300. Calculations are based on an annual average direct normal irradiation of 6.65 kWh/m^2^/day.

Next, we calculated the amount of electricity and clean water that could be produced by using 60% of the maximum power available from the E and 50% of the maximum available thermal energy from the W portions of the spectrum for each task to account for typical discrepancies between module efficiencies and the Shockley-Queisser limit^29, 30^, as well as other losses such as mirror imperfections. Thus, 60% of the maximum power shown in Fig. S3 was used for electricity generation. The maximum achievable temperature using the W spectrum was also calculated as a function of solar concentration to ensure that thermal desalination, which requires a minimum temperature of 121 °C, can be performed (Fig. S1A and B). From a 100 hectare of land and solar concentration of 20 with SJ solar cells (see Fig. 4), the daily availability for electricity and clean water are estimated to be 347 MWh and 744.3 thousand gal (Tgal) respectively (Table S8). The corresponding numbers for DJ solar cells (see Fig. 4) are 468 MWh and 270.0 Tgal. In contrast, if this same amount of food, electricity, and clean water were produced using independent land areas for each activity, we estimate the total land area needed to be about 180 hectare. For a solar concentration of 300, the estimated electricity generation using SJ jumps up to 387 MWh and 520 MWh for DJ cells; however, the impact on water purification is minimal. Note that only 50% of the land area is assumed to be covered by parabolic troughs or PV modules in all cases. See Electronic Supplementary Information for more details.

We also estimate the electricity generation from a 100 hectare land for plants that can thrive in 70% of photon intensity of the visible portion of the spectrum, such that a portion can be harvested as electricity as shown for Fig. 2C. For solar concentration of 20, the daily availability for electricity is estimated to be 510 MWh with SJ solar cells and 631 MWh for DJ solar cells. For higher solar concentration of 300, the estimated electricity generation using SJ cells is 551 MWh, and 684 MWh for DJ cells (see Table S8).

**Figure 5:**
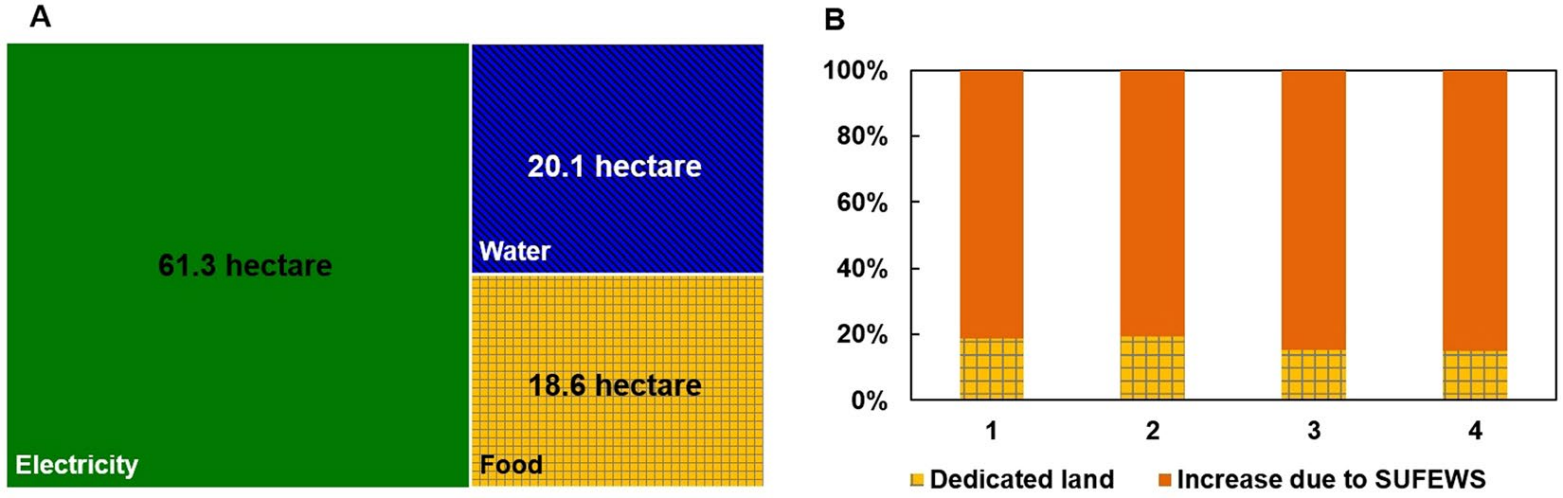
(**A**) Land area requirement to produce same electricity and water output of case 1 from separate dedicated lands. Note that when this is done, food production drops to only 18.6 hectare from the full 100 hectare of production achieved with SUFEWS. (**B**) Food production relative to a 100 hectare SUFEWS land for four different cases considered here.

The benefit of SUFEWS for food production is estimated for a 100 hectare of land area split and used for dedicated purposes of electricity generation and water production. To have the same electricity and water output of case 1, 61.3 hectare land area should be dedicated to electricity generation and 20.1 hectare for water production, which leaves 18.6 hectare land for food production (Fig. 5A). Without SUFEWS, food production is reduced by 80.7% to 84.9% relative to the full potential of the land as shown in Fig. 5B. Furthermore, due to the local availability of electricity and heat, water in SUFEWS farms will be more amenable to local water management in the form of improved irrigation (including drip irrigation) and collection/purification of runoff water from the farmland. Enough energy is available to purify water for local needs. This will have two benefits. First, irrigation as compared to rain-fed irrigation alone, increases crop yield and year-to-year predictability^2^. Given that ~25% of the worldwide farmland that is irrigated produces 33% of the world’s crops, we estimate that irrigation increases yield by about 48%^31^. The global implementation of SUFEWS across all current farmlands could deliver 32% more agricultural products, solely from the irrigation improvement. Second, runoff water from farmland will be collected and purified to decrease the need for clean water, as it can be recycled back to the field or lake, rivers, and other aquifers and, therefore, reduces the water footprint (Fig. 3). Also, nitrogen and phosphorous rich streams from the purification of the runoff water may be recycled, leading to reduction in fertilizer demand and avoiding algal blooms in aquifers receiving runoff water^2^. Alternatively, nutrient rich water streams can be sent for further downstream processing. Furthermore, for farmlands located near coastal areas, fresh water can be produced by desalination using energy from a given farm land area using SUFEWS; any surplus supply can be sent to adjoining urban areas.

## Conclusion

In conclusion, SUFEWS presents a feasible path to sustainably generate global electricity and fresh water at a local level without competing with food growth. By maximizing the utilization of the solar spectrum, SUFEWS makes the tremendous land area currently used for agriculture available for the co-production of electricity and thermal energy for water treatment, while *improving* crop yield due to absence of harmful shadows and maintenance of uniform light, and enhanced water generation and irrigation. The fresh water needed can be sourced from aquifers and oceans, which can be purified using SUFEWS prior to its use. Runoff water and nutrient laden waste streams from agricultural land can be reprocessed and recycled to the field to reduce demand for raw materials to produce fertilizers and reduce contamination to rivers and lakes responsible for damaging algal blooms and other ecological harm. The proposed system can create solar-power, FEW self-sufficient communities- a major step toward Full Earth preparedness. Furthermore, implementing SUFEWS across agricultural land areas could supply extra electricity and fresh water to the electric grid and water supply network to other areas in need, thus improving global resilience.

## Methods

Well-known process system analysis methods in conjunction with the commercial software ASPEN Plus v.8.8 and MATLAB were used to perform all of the material and energy balances and the maximum energy conversion efficiencies. The calculation details and results are provided in *Supplementary Information*.

## Electronic supplementary material

Supplementary Information
