# Supplementary material for: Directing solar photons to sustainably meet food, energy, and water needs
Source: Sci Rep. 2017 Jun 9;7:3133. doi: 10.1038/s41598-017-03437-x (PMC5466672; doi:10.1038/s41598-017-03437-x)
Supplement: Supplementary file 1 — Supplementary Information [file 41598_2017_3437_MOESM1_ESM.pdf]

Supplementary Information

Directing solar photons to sustainably meet food, energy, and water needs

Authors: Emre Gençer[1], Caleb Miskin[1], Xingshu Sun[2], M. Ryyan Khan[2], Peter Bermel[2], M. Ashraf Alam[2] and Rakesh Agrawal[1]*

Affiliations:
[1] School of Chemical Engineering, Purdue University, West Lafayette, IN-47907, USA

[2] School of Electrical and Computer Engineering, Purdue University, West Lafayette, IN-47907, USA

*Correspondence to: agrawalr@purdue.edu

1. Solar Spectrum Unbundling Design

Our objective is to determine how the solar spectrum can be split in an optimal fashion. We start by constructing the basic component of a spectral-splitting structure capable of transmitting and reflecting two separate wavelength ranges over a broad range of angles. While a quarter-wave stack offers some of these capabilities, a broad reflection range at infrared wavelengths also creates undesirable higher-order reflections in the visible spectrum. To get around this challenge, we have developed a global optimization approach for a multilayer filter design using an adjoint method for enhanced computational efficiency. We have shown that this design can be built and tested to work almost identically to experiment, as shown in Fig. S1A[1]. This basic design can also be scaled to reflect different cutoff wavelengths and can include multiple reflection ranges. Ilic et al. designed and built a 90 layer stack of SiO_2 and Ta_2O_5 that had nearly 90% transmission across the visible spectrum and high reflection for the infrared spectrum[1]. Similarly, suitable short-pass and band-pass dichroic mirrors can be used for this application. As a next step, we adjusted the cutoff wavelengths based on the lowest PV bandgap calculated and then calculated the optimal temperature of operation for a thermal collector based on the capability to do work (exergy). As shown in Fig. S1B, even modest concentrations of 20 suns and PV bandgaps around 0.8 eV allow optimal operation at around 675 K from W photons (all photons in solar spectrum with energy less than 0.8 eV). This heat is for water purification.

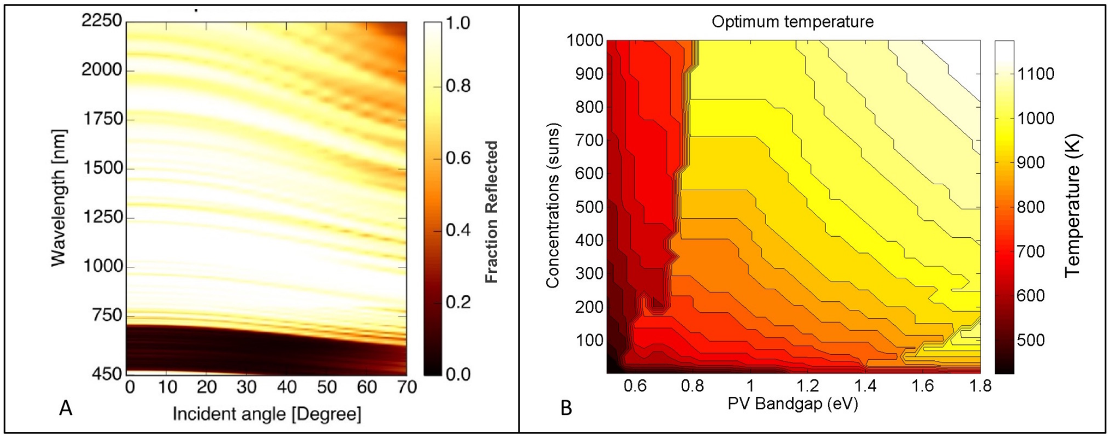

Fig. S1 A: Measurements of our prior experiments in spectral splitting, showing that a transparent window at visible wavelengths can exist in conjunction with a strongly reflecting region in infrared. The cutoff can be tuned to fall between 750 and 1,550 nm, depending on the application; B: Optimal operating temperature (in K) to maximize the potential to perform work (exergy) in purifying water or generating electricity, as a function of solar concentration and PV bandgap. At 20 suns, this corresponds to an operating temperature of 675 K.

1.1. Applicability and brief description of SUFEWS

In SUFEWS design, we do not simply transfer reflected light into shadowed areas. With the use of available dichroic short-pass polymeric films, 90% of the F photons are transmitted to the shadowed area. Some currently available examples include short-pass dichroic mirror films from 3M as Prestige 90 EX[2] and short-pass polymeric films that transmit 90% of F photons and reflect 90% of the E photons[3]. The reflected portion is composed of ~90% of direct light E photons in the solar beam. The shadow is basically deprived of E & W photons but contains F photons. A remaining piece of the puzzle is the design and fabrication of the optimal solar cells for the E photons. Indeed, our proposed solution is novel and quite likely to succeed.

Existing PV technologies and light harvesting farm assemblies like the ones used in urban farming or vertical farming can simultaneously allow the production of food and generation of electricity. However, such an integration has one fundamental disadvantage, which is that they do not benefit

from the entire solar spectrum and that is precisely the problem what we are hoping to overcome with our SUFEWS design.

In an alternative setup, solar energy is harnessed as electricity and then a portion is converted into light and the remaining electricity is supplied to the grid or used locally. The initial conversion step reduces the overall efficiency and it is a missed opportunity to benefit more from the solar irradiance. In SUFEWS design, the solar spectrum is carefully split in three zones that matches the requirement of the final utilization from each zone. F spectrum, provides photons in the wavelength range of less than 750 nm, which is what crops need for photosynthetic activity. E spectrum is determined from the remaining portion after the food production is guaranteed. The cutoff wavelength and achievable efficiency to generate electricity is rigorously calculated using methods described in the subsequent sections. Finally, the remaining portion of the solar spectrum (W) is dedicated to harness solar thermal energy, which is the best use of these low energy photons. Thus in urban farming or vertical farming cases, if solar energy is first converted to electricity using record efficiency Si modules then only 22.9% of solar energy would be harnessed as electricity. If the electricity were to be used to supply light for food, then the value of such a light cannot exceed 22.9% of the value of the solar spectrum. On the other hand, in our proposed idea, nearly 80 to 90% of the ~46% of photons with wavelength less than 750 nm will be transmitted and greater than ~37% of solar energy would be available for food growth. Additionally, we will have remaining photons for E and W usage. The advantage of our proposed solution is clear.

With the described three-way split, the entire spectrum is judiciously used for the production of FEW.

We have included practical losses in all of our calculations. First, we have assumed the deployment of SUFEWS on only 50% of the land area to allow spacing between units. Second, for electricity

generation, we have used 60% of the theoretical efficiency as the achievable efficiency that is a factor to account for practical losses such as reflective and thermal. Third, we have used 50% efficiency for the thermal energy recovery to account for the losses from the solar thermal energy harnessing. In short, we have accounted for all the losses.

2. SUFEWS Cases and Modeling Details

For the systems calculations we have considered two solar concentrations (C=20 and C=300) and two PV technologies (single junction (SJ) and double junction (DJ)) cases. For all cases, the F portion of the solar spectrum dedicated for growing food consists of all photons of $\lambda<\lambda_1$, where λ_1 is chosen to be 750 nm based on the portion of the spectrum needed for photosynthesis. λ_2 is optimized to achieve maximum sun-to-electricity efficiency from the remaining portion of the solar spectrum. The general concept is shown in Fig. S2. Solar energy is split into three portions namely F, E, and W. In the base case, the F portion is used for crop growth, the E portion is for electricity generation, and the W portion is for water treatment using thermal processes. The underlying assumption of the base case is that the entire F spectrum (<750nm) with full available intensity is needed for plant growth. In this case, the available energy directed to planting, electricity generation, and heat for water treatment is denoted as a, b, and c, respectively. In the case that the plants can thrive with partial intensity of the F fraction of the solar spectrum, the available energy in F is divided into a and d, where d is also used for electricity generation. This would particularly be true in very hot, arid climates where some shading would even be expected to be beneficial by reducing heat stress. A recent study has demonstrated that the nutritional content, inedible components of the biomass, and health index of wheat plants did not show significant difference under reduced intensity light (50% of the normal intensity) with wavelength in the range of 350 nm and 750 nm during seedling and grain filling stages[4]. In order to implement SUFEWS, more detailed studies for the entire planting season for wheat as well as for other wide array of

crops under different solar spectrum intensities will be needed. However, it is worth mentioning that during the day as a concentrator tracks the sun, the location of the shadow on the ground moves. Therefore, impact due to a potential reduction in the F spectrum intensity during transmission through the concentrator mirror will be somewhat mitigated.

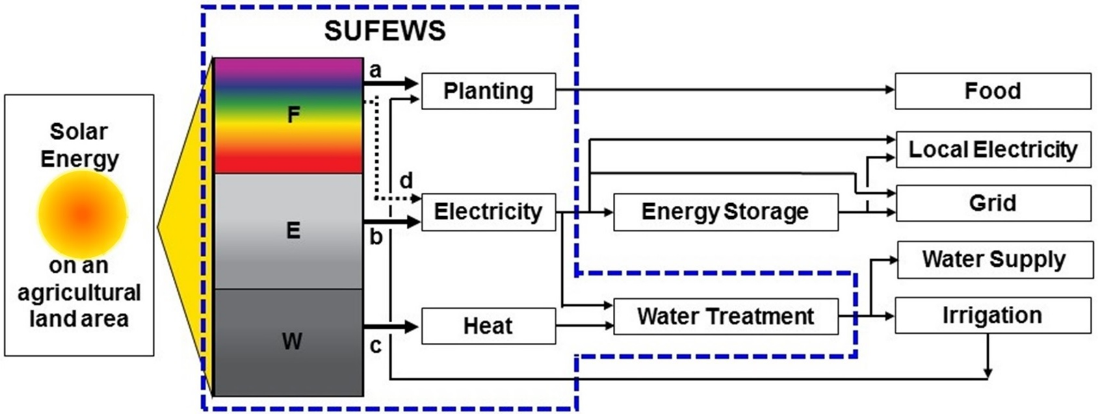

Fig. S2 General SUFEWS concept where solar spectrum is split into three portions andeach one is used where they can be optimally used.

The selective use of the solar spectrum in PV is not realized by PV modules. However, it can be achieved by our sophisticated multi-layer reflector design shown in Fig. 2. Electricity generation capacity by PV is calculated based on: i) an achievable PV efficiency depending on the cell configurations, see Table S3 for full list, which now includes both the efficiency based on the E portion of the spectrum (750 nm$<\lambda<\lambda_2$) and the full spectrum, ii) achievable efficiency is calculated based on 60% of the thermodynamic limit (maximum efficiency, Table S1) to account for losses, iii) as indicated in the main text and Supplementary Information, calculations are performed for an annual average direct normal irradiation of 6.65 kWh/m^2/day.

We would like to stress that our calculation methodology is consistent with the existing and emerging technologies. For example, under full-spectrum AM 1.5D, the Shockley-Queisser (SQ) limit for a single junction solar cell is 31%[5,6] and reported module efficiency for crystalline Si and GaAs solar cells are 22.9% and 24.1%, respectively.[6] These achieved values of efficiencies are in

the range of 73.9% to 77.7% of the maximum thermodynamic limit (SQ limit). Therefore, in our calculations, we first calculate the maximum achievable thermodynamic efficiency for the E photons and then take the achievable module efficiency to be 60% of this value. Thus, we account for all expected losses (such as 90% reflection from a dichroic mirror, etc.) in our power conversion efficiency calculation for solar cells operating using only the E photons. Thus we believe that the device efficiencies used in our calculations are justified and are very likely to be realized when practiced.

2.1. Conversion Efficiencies

The calculation procedure to estimate the recoverable electricity and thermal energy from the available energy in the photons not used for food (photons with wavelength greater than 750nm) is as follows.[7,8] First the maximum achievable efficiencies of SJ and DJ PV systems under solar concentration of 20 and 300 are calculated as described below. The determination of efficiencies is an optimization problem since the cut-off wavelength (λ_2) is a decision variable to maximize the electricity recovery from the remaining portion of the spectrum that is not used for food production. For the SJ case, the band gap of the solar cell that maximizes electricity generation is taken to be the cutoff energy for photons in the E range. Similarly, for the DJ solar cells, the lower band gap bottom cell provides us this value. Thus λ_2 is calculated here through maximization of electricity generation. This is done as electricity is the fastest growing component of energy that is used and it is the most versatile in meeting end energy needs.

2.2.General formulation for photovoltaic energy conversion within the energy-nexus system:

Solar source:

Photon flux from the Sun is given by $n_S(E)$. We use the standard spectrum for direct sunlight, i.e., AM1.5D. The sunlight contains 90% direct solar beam with a power of 0.9 kW/m^2. As described in the main text, a filter is used to redirect a part of the spectrum with $E \leq E_P$ (or, $\lambda \geq \lambda_1$ where $\lambda_1 \approx 750$ nm) to solar cells and thermal collectors while the rest of the spectrum passes through to the plants. Therefore, the solar flux incident on the solar cell device, $n_{IN}(E)$, in Fig. 2A is given by:

$$n_{In}(E) = \left(1 - A_P(E)\right) \times n_S(E) \times f_{conc}. \quad \text{(S1)}$$

Here, f_{Conc} is the solar concentration factor, and $A_P(E)$ is the portion of the spectrum related to the plants. We use ideal absorption profile defined as,

$$A_P(E) = \begin{cases} 0, \text{for } E < E_P \\ 1, \text{for } E \geq E_P \end{cases}. \quad \text{(S2)}$$

Radiation from a cell/device:

Under ideal considerations, we assume only radiative recombination in the solar cells. Now, photon flux from a blackbody is given by:

$$n_D(E, V) = \frac{(2\Omega_D / c^2 h^3)\, E^2}{\exp((E - \Delta\mu_D)/kT_D) - 1}, \quad \text{(S3)}$$

Here, T_D is the device temperature, Ω_D is the radiation angle, and $\Delta\mu_D (= qV)$ is the chemical potential. The device structure is such that both faces are transparent (i.e., bifacial see Fig. 2A) so

the unabsorbed sunlight can pass through the solar cell and be used for water purification. Hence, we set $\Omega_D = 4\pi$. Note that, the solar cell will only radiate above its bandgap. Thus the radiation spectrum at bias V would be given by $A(E)n_D(E,V)$, where $A(E)$ is the absorption (or, emission) coefficient.

Single-junction PV:

The net absorption (J_{SUN}) and radiation ($J_{rad}(V)$) from the single-junction (SJ) PV are given by,

$$J_{SUN} = q\int_0^{\infty} A_{SJ}(E)n_{in}(E)dE \tag{S4}$$

$$J_{rad}(V) = q\int_0^{\infty} A_{SJ}(E)n_D(E,V)dE \tag{S5}$$

Here, $A_{SJ}(E)$ is the absorption coefficient of the active layer of the device. From detailed particle balance [9], we can calculated the $J - V$ relationship as follows:

$$J(V) = J_{SUN} - J_{rad}(V) \tag{S6}$$

Now, we can find $P_{out}(V) = J\times V$ and $\max(P_{out}(V))$ from the above formulation for the SJ-PV.

Double-junction PV:

The top subcell of the double-junction (DJ) tandem PV receives the complete incident spectrum. Therefore, similar to the SJ-solar cell relationships, we can find $J - V$ for the top (t) subcell (bandgap $E_{g(t)}$),

$$J_{SUN(t)} = q\int_0^{\infty} A_t(E)n_{in}(E)dE \tag{S7}$$

$$J_{rad(t)}(V_t) = q\int_0^\infty A_t(E) n_D(E, V_t) dE. \tag{S8}$$

$$J_t(V_t) = J_{SUN(t)} - J_{rad(t)}(V_t) \tag{S9}$$

Now, the bottom subcell receives the part of the solar spectrum unabsorbed by the top subcell (i.e., $E < E_{g(t)}$). Thus, the $J - V$ relations for the bottom (b) subcell (bandgap $E_{g(b)}$) can be found as follows:

$$J_{SUN(b)} = q\int_0^\infty A_b(E)(1 - A_t(E)) n_{in}(E) dE, \tag{S10}$$

$$J_{rad(b)}(V_b) = q\int_0^\infty A_b(E) n_D(E, V_b) dE. \tag{S11}$$

$$J_b(V_b) = J_{SUN(b)} - J_{rad(b)}(V_b). \tag{S12}$$

Finally, the DJ series connected tandem characteristics can be found from, $J_{DJ}(V) = J_t(V_t) = J_b(V_b)$ with $V = V_t + V_b$. Now, the tandem cell is optimized for the band gap-pair (i.e., $opt(E_{g(t)}, E_{g(b)})$) to yield the maximum output.

By solving the optimization model described above, the maximum possible power conversion corresponding to only radiative recombination is calculated as a function of cutoff wavelength λ_2 for photons not used for growing food ($\lambda \geq 750$ nm) and results are shown in Fig. S3. Note that the results in Figure S3 are calculated for active area, whereas in actual operation only about 50% of the land area would be covered with SUFEWS. We account for this in subsequent calculations.

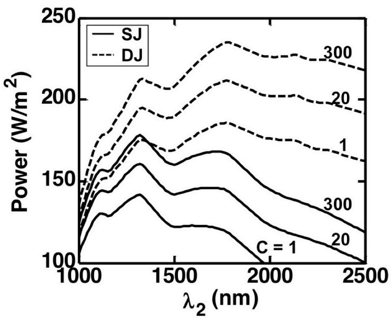

Fig. S3 Thermodynamic limit for electric power from E portion of spectrum at cutoff wavelength of λ_2. C is concentration factor.

Based on the fact that only photons with wavelengths 750 nm < λ < λ_2 are absorbed by the solar cell, we calculated the corresponding maximum possible efficiency (Table S1). For single junction (SJ) PV at solar concentrations of 20 and 300 the maximum efficiencies are 49.47% and 54.04%, respectively with corresponding cutoff wavelengths of 1315 nm and 1336 nm (Table S1). For double junction (DJ) PV at solar concentrations of 20 and 300 the maximum efficiencies are 53.34% and 59.26%, respectively with corresponding cutoff wavelengths of 1780 nm and 1780 nm. **Note that these efficiencies are based on the E portion of the spectrum (750 nm to λ_2) only.** The corresponding full spectrum efficiencies are also shown in Table S1 and should be achievable.

Table S1 Maximum achievable PV efficiencies based on both the E portion of the AM1.5D spectrum and full AM1.5D spectrum.

	Single Junction			Double Junction		
C	Max Efficiency (E photons)	Max Efficiency (Full Spectrum)	Cutoff Wavelength (λ_2)	Max Efficiency (E photons)	Max Efficiency (Full Spectrum)	Cutoff Wavelength (λ_2)
**20**	49.47%	17.9%	1315	53.34%	23.6%	1780
**300**	54.04%	19.9%	1336	59.26%	26.3%	1780

On the basis of calculated values of λ_2 corresponding to maximum achievable solar cell efficiencies, the E and W portions of the solar spectrum (Fig. 1B) are assigned and the corresponding power in each portion of the spectrum per hectare of land area are given in Table S2.

We have created eight cases listed in Table S2. Cases 1-4 utilize full intensity of the F spectrum for planting and cases 5-8 utilize 70% of the F spectrum for planting. The available solar energy for each case is given by a, b, c, and d as shown in Fig. S2 taking into account that the SUFEWS we consider here only covers 50% of a given land area.

Table S2 Available solar energy in each fraction based on AM1.5D solar spectra as defined in Fig. S2 taking into account the 50% land coverage reduction.

case	Photosynthesis Intensity	Conversion Technology	Solar Concentration	a	b	c	d
				[MW/hectare]			
1	100%	SJ	20	2.30	1.63	0.57	0
2	100%	DJ	20	2.30	1.99	0.21	0
3	100%	SJ	300	2.30	1.66	0.54	0
4	100%	DJ	300	2.30	1.99	0.21	0
5	70%	SJ	20	1.61	1.63	0.57	0.69
6	70%	DJ	20	1.61	1.99	0.21	0.69
7	70%	SJ	300	1.61	1.66	0.54	0.69
8	70%	DJ	300	1.61	1.99	0.21	0.69

The available solar energy for electricity generation is given by (b + d) for any case. Thermal energy is generated from c. Cases 1, 3, 5, and 7 utilize SJ, cases 2, 4, 6, and 8 utilize DJ PV technology. Cases 1, 2, 5 and 6, utilize solar concentration of 20 and cases 3, 4, 7, and 8 utilize solar concentration of 300.

For modeling, the achievable efficiencies (Table S3) are presumed to be 60% of the maximum efficiencies given in Table S1. This is chosen based of the fact that crystalline silicon PV modules today easily achieve 60% of the Shockley-Queisser limit. For example, under full-spectrum AM

1.5D, the Shockley-Queisser (SQ) limit for a single junction solar cell is 31%,[5] whereas the reported module efficiency for crystalline Si and GaAs solar cells are 22.9% and 24.1%, respectively[6]. These achieved values of efficiencies are in the range of 73.9% to 77.7% the maximum thermodynamic limit (SQ limit). Therefore, in our calculations, we first calculate the maximum achievable thermodynamic efficiency for the E photons and then take the reasonably achievable module efficiency to be 60% of this value, which is justified based on today's best technologies and likely to be realized when practiced. Thus, we have accounted for all expected losses (such as 90% reflection from a dichroic mirror, etc.) in our power conversion efficiency calculation for solar cells operating using only the E photons.

Table S3 Reasonably achievable PV efficiencies based on the E portion and full range of the solar spectrum.

	**Single Junction**			**Double Junction**		
**C**	**Max Efficiency (E photons)**	**Max Efficiency (Full Spectrum)**	**Cutoff Wavelength (λ_2)**	**Max Efficiency (E photons)**	**Max Efficiency (Full Spectrum)**	**Cutoff Wavelength (λ_2)**
**20**	29.68%	10.8%	1315	32.00%	14.2%	1780
**300**	32.42%	11.9%	1336	35.56%	15.8%	1780

The recoverable thermal energy from W spectrum is assumed to be 50% of the energy available in W spectrum or c in Table S2, which accounts for imperfect absorption, thermal losses to the environment, and other parasitic losses. As shown in Fig. S1 the achievable heat temperatures are greater than the temperature levels (121°C) required to run thermal desalination processes. Hence, all the available heat can be directed to generate steam and run a thermal desalination process such as multi stage flash (MSF) used for this study. It should be noted that since much higher temperatures are feasible, one could generate electric power using a solar thermal power cycle while rejecting heat at 121°C for MSF and further increase electricity production with SUFEWS;

however, this scenario was not considered here and in calculations. Using the listed assumptions and conversion values, the electricity generation capacity and recoverable thermal power from a hectare land is estimated and tabulated in Table S4.

The achievable PV efficiencies are optimized for E portion of the solar spectrum. In our calculations, for low intensity planting cases (5-8), we have again calculated the maximum possible PV efficiency for a wavelength range (λ) less than or equal to 750 nm using the same method described for the determination of the maximum PV efficiencies to convert the E portion of the spectrum. This maximum theoretical efficiency for the F portion of the spectrum is found to be 53.5% (27.3% on a total spectrum basis). In cases (5-8), we have assumed that the available energy (d in Fig. S2 and Table S2) from the F portion of the solar spectrum is converted to electricity with a PV efficiency of 32.1% (16.4% on a total spectrum basis), where again we have used 60% of the maximum SJ PV efficiency for the F portion of the spectrum. Again, we find that the presumed efficiencies are quite reasonable in comparison with standard modules in production today. The results are summarized in Table S4.

The corresponding per hectare daily electricity and thermal energy generation capacities of SUFEWS for an annual average direct normal irradiation (DNI) of 6.65 kWh/m^2/day (California or CA) are summarized in Table S5. For full intensity F spectrum cases 3.58 MWh to 5.24 MWh electricity can be generated daily per hectare with cogeneration of 0.76 MWh to 2.10 MWh thermal energy cogenerated. For reduced intensity plant growing scenarios, daily electricity generation can reach up to 6.88 MWh/hectare. These are exciting results and provide us with the potential to coproduce electricity and heat locally at a farm without impacting food production. Such coproduction allows the execution of energy intensive operations related to agricultural activities.

Table S4 Generated electricity and harnessed thermal power based on available energy for AM1.5D solar spectra as listed in Table S2 calculated using efficiencies from Table S3.

case	Electricity [MW/hectare]	Thermal Power [MW/hectare]
1	0.48	0.28
2	0.64	0.10
3	0.54	0.27
4	0.71	0.10
5	0.71	0.28
6	0.86	0.10
7	0.76	0.27
8	0.93	0.10

Table S5 Daily production capacity of SUFEWS in California calculated based on California's annual average DNI of 6.65 kWh/m^2/day.

case	Electricity [MWh/hectare·day]	Thermal Power [MWh/hectare·day]
1	3.58	2.10
2	4.72	0.76
3	3.98	2.00
4	5.24	0.76
5	5.22	2.10
6	6.35	0.76
7	5.62	2.00
8	6.88	0.76

The SUFEWS generation potential is also calculated for an annual average direct normal irradiation of 4.17 kWh/m^2/day (the average United States or US) and results are shown in Table S6. For full intensity F spectrum cases 2.24 MWh to 3.28 MWh electricity can be daily generated per hectare with 0.48 MWh to 1.31 MWh thermal energy cogenerated. For low intensity plant growing scenarios, daily electricity generation can reach up to 4.42 MWh/hectare.

Table S6 Daily production capacity of SUFEWS in the US calculated based on US's annual average DNI of 4.17 kWh/m^2/day.

case	Electricity [MWh/hectare·day]	Thermal Power [MWh/hectare·day]
1	2.24	1.31
2	2.96	0.48
3	2.49	1.25
4	3.28	0.48
5	3.27	1.31
6	3.98	0.48
7	3.52	1.25
8	4.31	0.48

3. Agricultural Land

Agricultural land as defined by The World Bank refers to the share of land area that is arable, under permanent crops, and under permanent pastures. Arable land includes land defined by the Food and Agriculture Organization of the United Nations as land under temporary crops (double-cropped areas are counted once), temporary meadows for mowing or for pasture, land under market or kitchen gardens, and land temporarily fallow. Land abandoned as a result of shifting cultivation is excluded. The agricultural land in the United States is 1,018.9 million acres or 412.3 million hectares, which corresponds to 44.3% of the total US land area. Total irrigated agricultural land area in the US is 62.4 million acres or 25.3 million hectares, which is 6.1% of the total agricultural area[10]. The amount of land devoted to farming and ranching in California was 25.5 million acres or 10.3 million hectares in 2014, which corresponds to 24.3% of the total California's land area[11].

2.1.Crop growth

The crop growth is dependent on many factors including geographical latitude, values of irradiation and ambient temperature. However, our system concept does not rely on any particular

crop or any other factors that affect the growth rate. For SUFEWS as well as in the comparative example in which land is used for only one purpose, the land is used to grow the same crop. Thus, the type of crop does not enter the equation and its specification is not needed. The extra 60% of land required in the comparative case is the additional land needed to produce the same quantity of electricity and water as SUFEWS is able to produce. The land usage reduction originates from the utilization of solar energy on a given land area for multiple purposes, i.e. generating electricity, harnessing solar thermal energy, and growing crops, unlike the current practice of using incident solar energy on a land area for only a single dedicated application. The underlying assumption supported by the publication of Zhu, *et al.* (2008)[12] is that C_3 and C_4 crops grow using the photosynthetic region of solar spectrum ($\lambda < 750$ nm). The presented system is especially important to meet local FEW needs in land-constrained areas like densely populated cities or remote communities without proper infrastructure.

4. Water purification technologies

There are two main methods for desalination: i) thermal processes and ii) membrane based technologies. While for the sake of completeness studying all the methods in detail is critical, only thermal multi stage flash desalination process is simulated in detail for this study. For cases of membrane based technology, reverse-osmosis is selected and its performance parameters are used from the literature.

4.1. Details of Desalination Simulation

The detailed simulation of multi stage flash (MSF) thermal desalination process shown in Fig. S6 is performed using Aspen Plus. The ELECNRTL as the base property method and STEAMNBS as the free-water method are used for the simulations. The salinity of the feed water is taken to be 0.040 kg NaCl/kg water (or 40 g NaCl/kg water). Ambient conditions of 35°C and 1 atm (1.013

bar) are used. Elec Wizard in Aspen Plus is run to accurately predict association and dissociation of Na^+ and Cl^- ions in water solution. An MSF desalination process consisting of 16 heat recovery stages and 3 heat rejection staged is considered for this study. As shown in Fig. S6, the saline water is first pumped to 3 bar and fed to the heat rejection section.

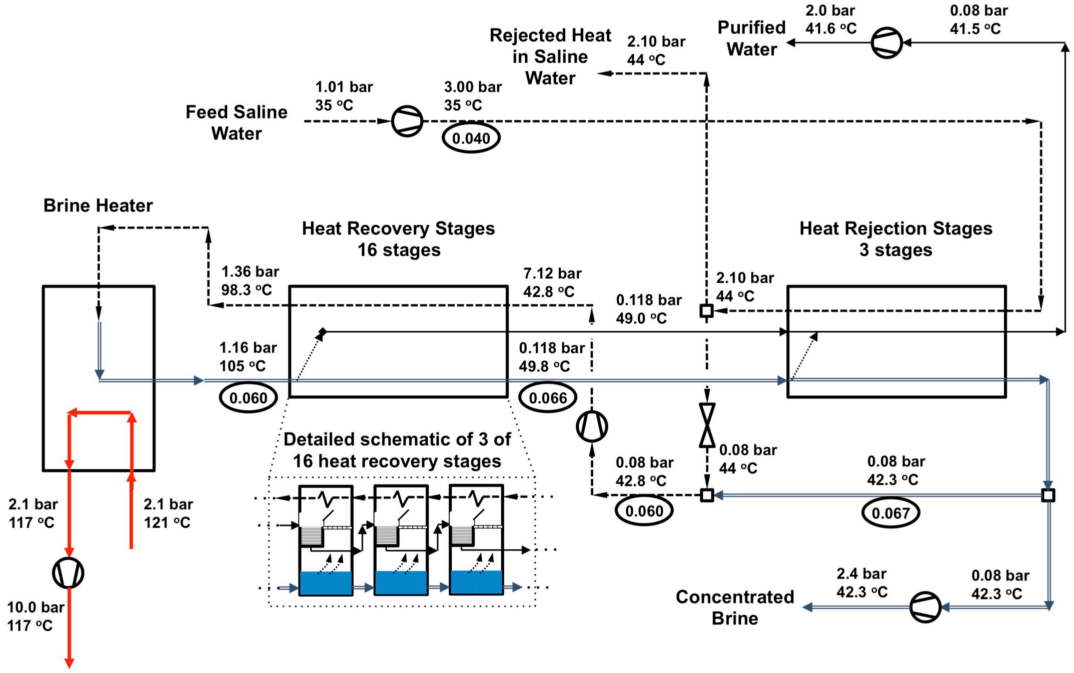

Fig. S4 A multi stage flash (MSF) desalination process flowsheet. Steam or a hot stream will be provided from W portion of the spectrum. Numbers in ellipses (⬭) are salinity (kg NaCl/kg of water).

A portion of the outlet saline water at 2.1 bar and 44°C is rejected, the remaining portion is depressurized to mix with the recycle brine. The mixed stream reaching salinity of 0.060 kg/kg water is pumped to 7.12 bar, to the inlet condition of the heat recovery stages. The pressurized brine and heated brine are fed counter current to the heat recovery stages. The discharge conditions of pressurized brine stream are 1.36 bar and 98.3°C. The discharged brine stream is heated to 105°C at 1.16 bar in the brine heater against steam at 121°C and 2.1 bar. Fresh water is produced at each stage of the heat recovery section according to the equilibrium vapor fraction of stage's condition. The discharge brine reaches a salinity of 0.066 kg/kg water at 0.118 bar and 49.8°C.

Following the heat rejection stages, a portion of the brine is rejected at 2.4 bar and 42.3^{o}C and the remaining portion is recycled and mixed with incoming brine at 0.08 bar and 42.3^{o}C. For 1000 kg/hr of saline water feed 134.6 kg/hr of fresh water is produced consuming 10.0 kWh thermal energy and 0.41 kWh electricity. Thus, to produce 1m^3 (or 264.2 gal) of fresh water, the simulated MSF process requires 74.5 kWh thermal energy and 4.1 kWh electricity.

Table S7 Energy requirement of producing 1m^3 (264.2 gal) fresh water

	**Thermal Energy**	**Electricity**
	[kWh]	[kWh]
**MSF**	74.5	4.1
**RO**	-	3-10 [13]

Table S8 Daily electricity and fresh water generation capacity for a hectare of agricultural land area in California using MSF desalination for water production calculated based on California's annual average DNI of 6.65 kWh/m^2/day.

	Electricity	Fresh Water	
**case**	**[MWh/hectare·day]**	**[m^3/hectare·day]**	**[gal/hectare·day]**
1	3.47	28.17	7443.37
2	4.68	10.22	2700.01
3	3.87	26.79	7077.69
4	5.20	10.22	2700.01
5	5.10	28.17	7443.37
6	6.31	10.22	2700.01
7	5.51	26.79	7077.69
8	6.84	10.22	2700.01

Table S9 Daily electricity and fresh water generation capacity for a hectare of agricultural land area in the US using MSF desalination for water production calculated based on US's annual average DNI of 4.17 kWh/m^2/day.

case	Remaining Electricity [MWh/hectare·day]	Fresh Water [m^3/hectare·day]	[gal/hectare·day]
1	2.17	17.65	4662.29
2	2.93	6.40	1691.20
3	2.42	16.78	4433.24
4	3.26	6.40	1691.20
5	3.20	17.65	4662.29
6	3.95	6.40	1691.20
7	3.45	16.78	4433.24
8	4.28	6.40	1691.20

Table S10 Daily fresh water generation capacity of a hectare of agricultural land area in California using MSF and RO for water production calculated based on California's annual average DNI of 6.65 kWh/m^2/day. The entire cogenerated electricity is used for water purification.

case	Fresh Water [m^3/hectare·day]	[gal/hectare·day]
1	721.42	190,599.28
2	945.39	249,772.05
3	800.44	211,475.11
4	1050.36	277,505.12
5	1048.86	277,109.63
6	1272.83	336,282.40
7	1127.88	297,985.46
8	1377.80	364,015.47

Table S11 Daily fresh water generation capacity of a hectare of agricultural land area in the US using MSF and RO for water production calculated based on US's annual average DNI of 4.17 kWh/m^2/day. The entire cogenerated electricity is used for water purification.

	Fresh Water	
**case**	**[m^3/hectare·day]**	**[gal/hectare·day]**
1	451.87	119,385.37
2	592.16	156,449.32
3	501.37	132,461.33
4	657.91	173,820.44
5	656.97	173,572.72
6	797.26	210,636.67
7	706.47	186,648.68
8	863.01	228,007.79

5. Estimation of Land Utilization Advantage of SUFEWS

To demonstrate the benefit of SUFEWS for land area usage, we have calculated the dedicated land area needed to generate the same amount of electricity and produce the same amount of fresh water using the entire solar spectrum. The land area remaining for food production is calculated for a total land area of 100 hectare. The total land coverage is assumed to be 50% for solar PV installation as well as solar thermal energy collection. To have a proper comparison, the electricity generation is calculated by using the maximum efficiencies (Table S12) calculated in the previous sections of the Supplementary information. Total electricity generation and corresponding overall electricity generation efficiencies (based on the entire solar spectrum) for cases 1-4 (described in Table S2) are given in Table S13.

Table S12 The available energy for electricity production from F and W portions of solar spectrum as described in Fig. S2; b refers to the available energy in E portion and d refers to the available energy for electricity production in F portion of solar spectrum. Conversion efficiencies are 60% of the maximum efficiency as given in Section 2.2.

	Available Energy		Conversion Efficiency	
	b	d	b	d
case	[MW/hectare]			
1	1.63	2.3	29.7%	32.1%
2	1.99	2.3	32.0%	32.1%
3	1.66	2.3	32.4%	32.1%
4	1.99	2.3	35.6%	32.1%

Table S13 The total electricity generated from F and E portions of solar spectrum using the data of Table S12. The overall efficiency refers to the output electricity compared to the entire available energy.

case	Generated electricity [MW/hectare]	Overall conversion efficiency
1	1.22	27.2%
2	1.38	30.6%
3	1.28	28.4%
4	1.45	32.1%

The dedicated land area requirements for electricity generation and fresh water production are calculated using power generation capacity given in Table S13 and thermal power need given in Table S5.

Table S14 Summary of land area utilization for separate electricity and water production, and the remaining land area from a total of 100 hectare to produce food.

	Dedicated land area		Remaining land area for
	Electricity	Water	Food
	(hectare)	(hectare)	(hectare)
1	61.3	20.1	18.6
2	73.5	7.3	19.3
3	65.4	19.2	15.4
4	77.6	7.3	15.1

Results are listed in Table S14 and shown in Fig. S5. To have the same electricity output of case 1, 61.3 hectare land needs to be dedicated for power generation. For the same water production an additional 20.1 hectare land is required. For a scenario of 100 hectare available land, 18.6 hectare land remains for food production, which corresponds to 81.4% reduction in food production compared to a land using the proposed SUFEWS.

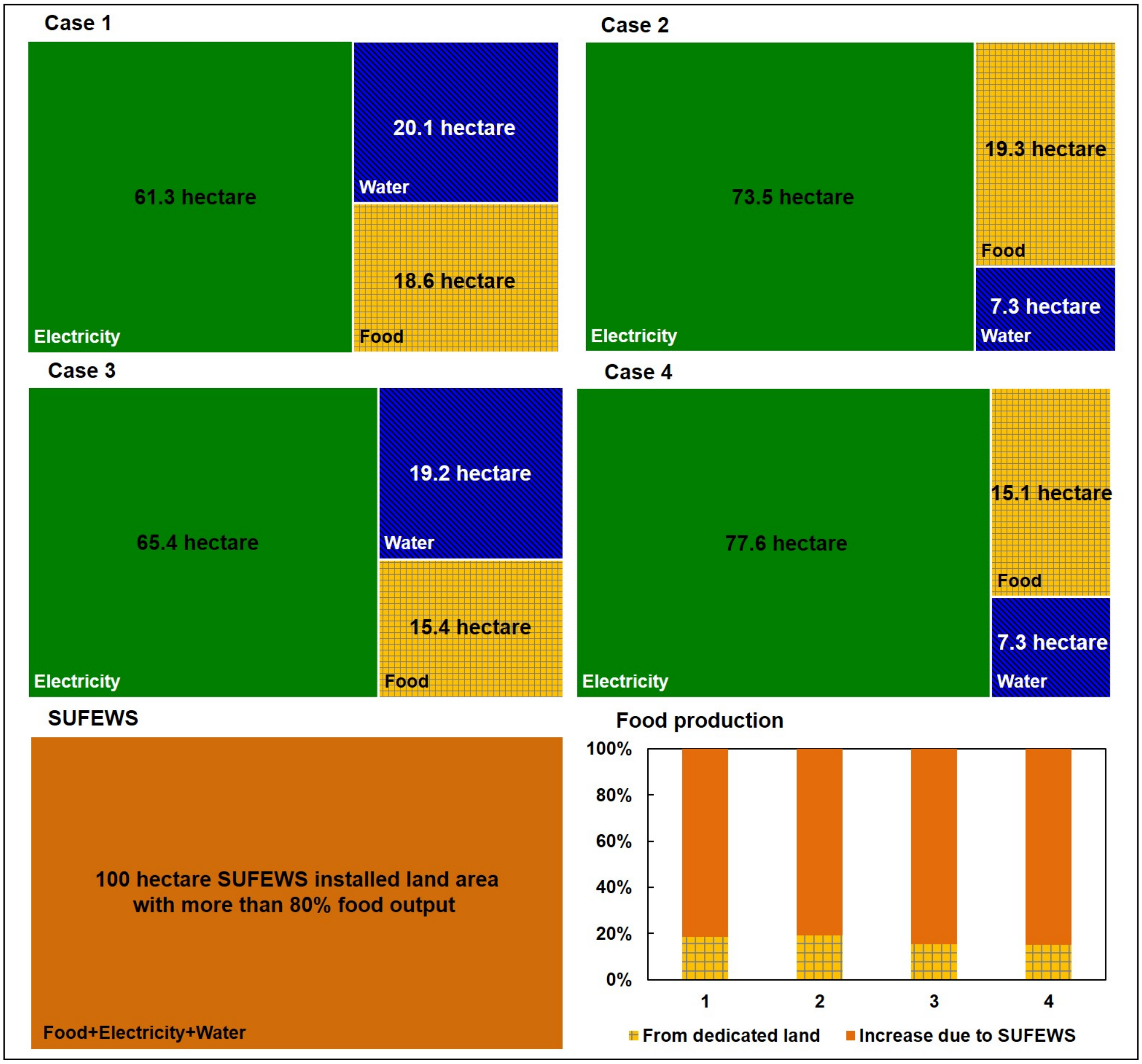

Figure S5 Summary of land utilization results for a 100 hectare land area that produces same electricity and water output using dedicated portions of the land. The remaining land area for food production and the relative food production with and without SUFEWS system. The four cases for SUFEWS are summarized in Table S2.

References

1. Ilic, O. *et al.* Tailoring high-temperature radiation and the resurrection of the incandescent source. *Nat. Nanotechnol.* 1–21 (2016). doi:10.1038/nnano.2015.309

2. 3M. *3M Sun Control Film Prestige 90 Exterior*.

3. Weber, M. F. Giant Birefringent Optics in Multilayer Polymer Mirrors. *Science (80-.).* **287,** 2451–2456 (2000).

4. Dong, C., Fu, Y., Liu, G. & Liu, H. Low light intensity effects on the growth, photosynthetic characteristics, antioxidant capacity, yield and quality of wheat (Triticum aestivum L.) at different growth stages in BLSS. *Adv. Sp. Res.* **53,** 1557–1566 (2014).

5. Green, M. A. & others. *Third generation photovoltaics*. (Springer, 2006).

6. Green, M. A., Emery, K., Hishikawa, Y., Warta, W. & Dunlop, E. D. Solar cell efficiency tables (Version 45). *Prog. Photovoltaics Res. Appl.* 1–9 (2015). doi:10.1002/pip

7. Ryyan Khan, M. & Alam, M. A. Thermodynamic limit of bifacial double-junction tandem solar cells. *Appl. Phys. Lett.* **107,** 223502 (2015).

8. Khan, M. R., Bermel, P. & Alam, M. A. Thermodynamic limits of solar cells with non-ideal optical response. *Conf. Rec. IEEE Photovolt. Spec. Conf.* 1036–1040 (2013). doi:10.1109/PVSC.2013.6744318

9. Shockley, W. & Queisser, H. J. Detailed Balance Limit of Efficiency of p-n Junction Solar Cells. *J. Appl. Phys.* **32,** 510–519 (1961).

10. Maupin, M. A. *et al. Estimated Use of Water in the United States in 2010: U.S. Geological Survey Circular 1405*. (2014). doi:http://dx.doi.org/10.3133/cir1405

11. Schwarzenegger, A., Chrisman, M., Agency, N. R. & Luther, B. California Farmland. (2008).

12. Zhu, X. G., Long, S. P. & Ort, D. R. What is the maximum efficiency with which photosynthesis can convert solar energy into biomass? *Curr. Opin. Biotechnol.* **19,** 153–159 (2008).

13. Charcosset, C. A review of membrane processes and renewable energies for desalination. *Desalination* **245,** 214–231 (2009).
